# Supplementary material for: Lifestyle habits associated with elevated depressive symptoms among healthcare workers during the COVID-19 pandemic
Source: PLOS Ment Health. 2025 Aug 21;2(8):e0000393. doi: 10.1371/journal.pmen.0000393 (PMC12798239; doi:10.1371/journal.pmen.0000393)
Supplement: Attachment — Submitted filename: Response to Reviewers 2025.5.19.docx [file pmen.0000393.s004.docx]

Responses to reviewers:

Reviewer #1: 

I would like to thank you for giving me the opportunity to review the manuscript entitled “Lifestyle habits associated with elevated depressive symptoms among healthcare workers during the COVID-19 pandemic”. The manuscript presents an interesting and potentially valuable study.

Thank you for allowing us to improve our paper. All of your guidance and important suggestions were carefully considered, and we had a very good experience. We would answer your questions one by one. Response content is highlighted in blue. In addition, revisions to the manuscript are highlighted in red.

However, it should be carefully revised. Please find my comments:

- Introduction: While the introduction highlights the pandemic's impact, it could better integrate findings from existing global studies to situate the research within the broader literature. For instance, comparisons with international CES-D studies would provide deeper context.

Answer: Thank you for valuable advice. We explained them in the introduction section.

Introduction section

Before

Based on this background, we thought that the significant changes in people's lifestyles due to COVID-19 may have made healthcare workers, who were at the forefront of COVID-19 treatment, more susceptible to depression.

After

Based on this background, we thought that the significant changes in people's lifestyles due to COVID-19 may have made healthcare workers, who were at the forefront of COVID-19 treatment, more susceptible to depression.

While previous studies in countries such as the United States and China have demonstrated an increase in depressive symptoms during the COVID-19 pandemic using the CES-D scale, targeting the general public [19, 20], and studies of healthcare workers have also shown similar trends, but several reports [21, 22] are limited to certain occupations such as residents or nurses, and there are few studies [23] that investigated many occupations such as doctors, nurses, physical therapists, and clerks. Additionally, our institution has conducted similar research examining depressive symptoms during the pandemic [11]. However, there has been no study that specifically identifies which lifestyle changes, known risk factors for depression, are particularly important within the context of lifestyle changes brought about by the pandemic. Furthermore, to the best of our knowledge, there are few studies that have investigated CES-D scores within the same population before and after the pandemic.

From this perspective, we believe it is highly meaningful to contribute to the existing literature by incorporating a new focus on "lifestyle" changes and conducting a study that compares the pre- and post-pandemic periods within the same population over time. This approach adds a valuable new perspective to the body of work and enriches our understanding of the impact of lifestyle changes on depressive symptoms during the pandemic.

[19] Alzueta E, Podhajsky S, Zhao Q, Tapert S, Thompson W, de Zambotti M, et al. Risk for depression tripled during the COVID-19 pandemic in emerging adults followed for the last 8 years. PSYCHOLOGICAL MEDICINE. 2023;53(5):2156-63.

[20] Peng S, Lai X, Du Y, Li Y, Tian K, Gan Y. Prevalence and Associated Factors for Depressive Symptomatology in Chinese Adults During COVID-19 Epidemic. FRONTIERS IN PSYCHOLOGY. 2020;11.

[21] Long X, Zhang J, Chen J, Shi L, Li X, Yi W, et al. Analyzing the influence of COVID-19 epidemic on the employment intention of resident physicians in China. JOURNAL OF INFECTION IN DEVELOPING COUNTRIES. 2022;16(6):1009-15.

[22] Song M, Park S, Park S. Factors Influencing Frontline Screening Nurses' Depression as a Long-Term Impact of COVID-19. WESTERN JOURNAL OF NURSING RESEARCH. 2023;45(6):554-61.

[23] Hoffmann S, Schulze S, Loeffler A, Becker J, Hufert F, Gremmels H, et al. Did the prevalence of depressive symptoms change during the COVID-19 pandemic? A multilevel analysis on longitudinal data from healthcare workers. INTERNATIONAL JOURNAL OF SOCIAL PSYCHIATRY. 2024;70(1):87-98.

[11] Katsuta N, Ito K, Fukuda H, Seyama K, Hori S, Shida Y, et al. Elevated depressive symptoms among newer and younger healthcare workers in Japan during the COVID-19 pandemic. Neuropsychopharmacol Rep 2021;41:544–7.

- The hypothesis could be more explicit regarding which specific lifestyle habits (e.g., physical inactivity, smoking) were expected to associate with CES-D positivity.

Answer:

Thank you for valuable advice. We briefly explained it in the introduction section.

Introduction section

Before

Therefore, we tested this hypothesis by comparing the CES-D results and lifestyle habits of healthcare workers before and after the COVID-19 pandemic.

After

We also hypothesized that physical inactivity which may have occurred under such behavioral restrictions would have had a particularly strong impact on CES-D positivity. Therefore, we tested this hypothesis by comparing the CES-D results and lifestyle habits of healthcare workers before and after the COVID-19 pandemic.

- Method: The description of the lifestyle questionnaire lacks specificity. Supplementary figures are referenced but should also be briefly described in the main text.

Answer: Thank you for valuable advice. Now we briefly described the content of the questionnaire in the method section.

Methods section

Before

“It contains the following items: (1) lifestyle, (2) medical history, (3) current illness history, (4) family history, (5) subjective symptoms, (6) lifestyle checks (diet, exercise, stress management, working hours, work performance, etc.), and (7) specific health check-related (including drinking habits).”

After

We collected the following information through a structured questionnaire conducted during routine occupational health check-ups:
(1) living situation (e.g., living alone or with family),
(2) medical history (presence or absence of past illnesses),
(3) current health conditions (e.g., hypertension, diabetes, cardiovascular diseases),
(4) family history of lifestyle-related diseases (e.g., hypertension, diabetes, cardiovascular diseases),
(5) subjective symptoms (e.g., headache, chest pain),
(6) lifestyle-related factors including smoking, presence of back pain or stress, dietary and exercise habits, time spent on visual display terminal (VDT) work, sleep duration, and information processing ability,
(7) items based on Japan’s Specific Health Checkups, such as adequacy of sleep, alcohol consumption, eating speed, and walking speed.

- Clarify how questions were validated for relevance to depressive symptoms.

Answer: Thank you for this important suggestion. The lifestyle questionnaire was developed for general purposes, not specifically for depression. Thus, some irrelevant questions are also included. However, we did not judge each item’s relevance to depressive symptoms in the viewpoint of causal relationship. We simply examined numerical relationship of each score and CES-D, without considering their causal relationship. As a result, only relevant lifestyles showed numerical relationship with depression, and we discussed the role of these lifestyles. We briefly explained in the methods section.

Methods section

Before

This questionnaire was created at our facility and has been used every year for health checkups.

After

This questionnaire was created at our facility and has been used every year for health checkups. The lifestyle questionnaire was developed for general purposes, not specifically for depression. Thus, some irrelevant questions are also included. In this study, however, we did not judge each item’s relevance to depressive symptoms in the viewpoint of causal relationship and used all items for the analysis. We simply examined numerical relationship of each score and CES-D, without considering their causal relationship.

- Results: Highlight key logistic regression findings directly in the text rather than relying heavily on figures.

Answer: Thank you for this important suggestion. As a result of the main logistic regression analysis, the questions associated with a positive CES-D in 2019 were "Q30 (Have you gained more than 10 kg from your weight at age 20?)" and "Q35 (What is the amount of alcohol you consume per day on drinking days?)," and the related questions in 2022 were "Q3 (Do you smoke cigarettes habitually?)" and "Q18 (How many steps do you take on average per day?)." The questions with the highest odds ratios in 2019 and 2022 were related to "Q5 (Are you good at controlling stress)," "Q36 (Does sleep helps you feel well rested?)," and "Q4 (Do you feel stressed every day?)," so we had added these points and made them clear in the text.

Results section

Before

Subsequently, we analyzed the factors expected to worsen the mental state of employees during the COVID-19 pandemic. For the employees in 2022 who also had paired data of negative CES-D for those in 2019 (total 1,872), logistic regression analysis was performed using binary category as the outcome variable (i.e., CES-D positive findings in 2022), and similar factors (single regression, correlation with a P value of < .2) were selected as the dependent variables. A forest plot was created for items that were significantly associated (P < .05) (Figure 3).

After

Subsequently, we analyzed the factors expected to worsen the mental state of employees during the COVID-19 pandemic. For the employees in 2022 who also had paired data of negative CES-D for those in 2019 (total 1,872), logistic regression analysis was performed using binary category as the outcome variable (i.e., CES-D positive findings in 2022), and similar factors (single regression, correlation with a P value of < .2) were selected as the dependent variables. A forest plot was created for items that were significantly associated (P < .05) (Figure 3). Questions related to “Q5 (Stress control),” “Q4 (To feel stressed)” and “Q36 (To sleep well),” had the highest odds ratios

- The manuscript notes a higher prevalence of depressive symptoms among females. Discuss this finding in more detail, including potential occupational or societal influences.

Answer: Thank you for this important suggestion. In terms of occupations in Japan, the large proportion of women among nurses is one of the factors that might explain the high prevalence of depressive symptoms among women. Nurses frequently come into contact with patients, and it is likely that the psychological burden of contacting patients during these emerging infectious diseases will increase. In addition, in the current situation where medical treatment is unpredictable, nurses had no choice but to follow the doctor's instructions, and the lack of discretion in work during a pandemic might also be contributing to the worsening of depressive symptoms. We had added the following reference paper and added this explanation to the discussion part of the text.

Discussion section

Before

This result is similar to reports from other facilities, reaffirming the importance of mental care for nurses.

After

This result is similar to reports from other facilities, reaffirming the importance of mental care for nurses.

In terms of occupation in Japan, the high proportion of women among nurses is considered to be one of the factors contributing to the higher prevalence of depressive symptoms observed in women. This may be largely attributable to biological sex differences, as the prevalence of depression is generally higher in women than in men. Additionally, because nurses frequently interact with patients, they may experience greater psychological burden during outbreaks of emerging infectious diseases. Furthermore, in clinical settings where future prospects are uncertain, nurses often have no choice but to follow physicians’ instructions. Under pandemic conditions, this lack of autonomy in their duties may also contribute to the worsening of depressive symptoms [31].

[31] Albert PR. Why is depression more prevalent in women? J Psychiatry Neurosci. 2015;40(4):219-21.

- Discussion: Expand the discussion on how specific factors like smoking and physical inactivity (e.g., <4,000 steps) contribute to mental health disparities. Are these findings consistent with pre-pandemic data?

Answer: Thank you for this important suggestion. Smoking and physical inactivity would affect the deterioration of mental health, and quitting smoking and exercising habits contribute to improving mental health, as stated in the cited literature, and we believe this is consistent with data from before the pandemic. It was generally known that smokers are more likely to develop depressive symptoms than non-smokers. Nicotine might seem to reduce stress in the short term, but in the long term it could affect the regulation of neurotransmitters (serotonin and dopamine), which can worsen mental health. Many studies have shown that exercise has an antidepressant effect, and physical inactivity is known to increase the risk of depressive symptoms and anxiety. We had added these explanations to the discussion part of the text. We have made revisions to the two discussion sections.

Discussion section

Before

（1）Because “Q18 (Less than 4,000 steps)” was associated with the CES-D positive in　2022, it should be necessary for all Japanese citizens to make an effort to avoid reducing the number of steps taken daily even if all citizens were forced to adopt the “new lifestyle.”

（2）Additionally, regarding “Q3 (To smoke cigarettes),” which was associated with the CES-D positive in 2022, the association between smoking and the increased risk of COVID-19 deaths [41] was well known in Japan. Thus, smokers might have been more susceptible to stress during the COVID-19 pandemic.

After

（1）Because “Q18 (Less than 4,000 steps)” was associated with the CES-D positivity in 2022, it should be necessary for all Japanese citizens to make an effort to avoid reducing the number of steps taken daily even if all citizens were forced to adopt the “new lifestyle.” Many studies have shown that exercise has an antidepressant effect, and physical inactivity is known to increase the risk of depressive symptoms and anxiety［12］.

[12]	Kunugi H. Depression and lifestyle: Focusing on nutrition, exercise, and their possible relevance to molecular mechanisms. Psychiatry Clin Neurosci 2023;77:420–33.

（2）Additionally, regarding “Q3 (To smoke cigarettes),” which was associated with the CES-D positivity in 2022, the association between smoking and the increased risk of COVID-19 deaths [41] was well known in Japan. Thus, smokers might have been more susceptible to stress during the COVID-19 pandemic. And it is generally known that smokers are more likely to develop depressive symptoms compared to non-smokers.［41］While nicotine may provide short-term stress relief, in the long term, it can affect the regulation of neurotransmitters such as serotonin and dopamine, potentially worsening mental health.

- Discuss other issues can be influence depressive symptoms among healthcare workers. The following article can be used:
1. <https://www.frontiersin.org/journals/public-health/articles/10.3389/fpubh.2022.1034624/full>
2. <https://www.mdpi.com/1660-4601/18/23/12548>

Answer: Thank you very much for suggesting important literature related to this study.

The report on ICU nurses [1] described experiences of feeling "distance from holistic nursing" (difficulty in having a human relationship with patients due to infection control measures, problems in the allocation of nursing care and professional care), as well as dissatisfaction with "organizational inefficiency" (lack of support for nurses in the organization, problems with the supply system of supplies, inefficiency in management, etc.). We also considered that these were psychological burdens that influenced depressive symptoms. In addition, the report on Iranian nurses during the COVID-19 pandemic [2] described the importance of employment stability in providing high-quality nursing care and the need to work to prevent the formulation and implementation of short-term and unstable employment contracts. It was suggested that instability in economic aspects such as employment contracts may affect depressive symptoms. After adding these two papers, the above explanation was added to the main text.

1. Nikbakht Nasrabadi A, Abbasi S, Mardani A, Maleki M, Vlaisavljevic Z. Experiences of intensive care unit nurses working with COVID-19 patients: A systematic review and meta-synthesis of qualitative studies. Front Public Health. 2022;10:1034624.
2. Maleki M, Mardani A, Vaismoradi M. Insecure Employment Contracts during the COVID-19 Pandemic and the Need for Participation in Policy Making. Int J Environ Res Public Health. 2021;18(23).

Discussion section

Before

Under pandemic conditions, this lack of autonomy in their duties may also contribute to the worsening of depressive symptoms [31].

After

Under pandemic conditions, this lack of autonomy in their duties may also contribute to the worsening of depressive symptoms [31].

In a report on ICU nurses , it is stated that experiences of "distance from holistic nursing" (the difficulty in maintaining human connections with patients due to infection control measures, issues with the allocation of nursing care, and problems in specialized care) and dissatisfaction with "organizational inefficiency" (lack of support for nurses, issues with the supply of resources, and inefficiencies in management) were reported. These factors may have contributed to psychological stress, which in turn could have influenced the development of depressive symptoms.[32]
Additionally, a report on nurses in Iran during the COVID-19 pandemic highlights the importance of employment stability in providing high-quality nursing care, emphasizing the need to advocate for the prevention of short-term and unstable employment contracts. The report suggests that instability in economic aspects, such as employment contracts, may affect depressive symptoms.[33]

[32] Nikbakht Nasrabadi A, Abbasi S, Mardani A, Maleki M, Vlaisavljevic Z. Experiences of intensive care unit nurses working with COVID-19 patients: A systematic review and meta-synthesis of qualitative studies. Front Public Health. 2022;10:1034624.

[33] Maleki M, Mardani A, Vaismoradi M. Insecure Employment Contracts during the COVID-19 Pandemic and the Need for Participation in Policy Making. Int J Environ Res Public Health. 2021;18(23).

- Provide more actionable recommendations for addressing CES-D positivity based on the findings. For example, what workplace changes could mitigate factors like high working hours or lack of physical activity?

Answer: Measures to improve long working hours in the workplace include improving work efficiency and proper division of labor, reviewing working hours (introducing a flextime system, etc.), and strengthening mental health support (implementing stress checks and mental health training, etc.).

Measures to improve the lack of physical activity include improving employee benefits to promote exercise, such as setting up fitness facilities, and creating opportunities for exercise at work, such as introducing standing desks. These practical measures have been added to the text. We have made revisions to the two discussion sections.

Discussion section

Before

（1）Moreover, a study targeting white-collar workers in Japan indicated that those who worked 50 hours or more per week had a higher risk for developing mental illness (odds ratio: 1.36) [47].

（2）Because “Q18 (Less than 4,000 steps)” was associated with the CES-D positive in 2022, it should be necessary for all Japanese citizens to make an effort to avoid reducing the number of steps taken daily even if all citizens were forced to adopt the “new lifestyle.” Many studies have shown that exercise has an antidepressant effect, and physical inactivity is known to increase the risk of depressive symptoms and anxiety [12].

After

（1）Moreover, a study targeting white-collar workers in Japan indicated that those who worked 50 hours or more per week had a higher risk for developing mental illness (odds ratio: 1.36) [47]. Possible measures to improve long working hours in the workplace include improving work efficiency and proper task distribution, reviewing working hours (such as introducing a flexible work schedule), and strengthening mental health support (such as implementing stress checks and providing mental health training).

（2）Because “Q18 (Less than 4,000 steps)” was associated with the CES-D positivity in 2022, it should be necessary for all Japanese citizens to make an effort to avoid reducing the number of steps taken daily even if all citizens were forced to adopt the “new lifestyle.” Many studies have shown that exercise has an antidepressant effect, and physical inactivity is known to increase the risk of depressive symptoms and anxiety [12]. As measures to address the lack of physical activity, we consider enhancing employee benefits to promote exercise, such as providing fitness facilities, and creating opportunities for physical activity during work, such as introducing standing desks.

Reviewer #2: The manuscript, "Lifestyle habits associated with elevated depressive symptoms among healthcare workers during the COVID-19 pandemic" does not meet the editorial criteria for publication. Although this is original research, the overall similarity percentage is higher than expected (26%) according to the Turnitin software report.

Thank you for pointing this out. Although we were aware that self-plagiarism should be avoided, in some sentences, our descriptions resembled those in our previous report analyzing overlapping dataset. This has caused relatively high similarity index. Now, we carefully checked the overlap with previous manuscript and by our check, similarity percentage is 16% according to the Turnitin software.

It is observed that there is no logical coherence between the title, aim, results and research design, it is stated that it is a retrospective study and in other parts of the manuscript it is stated that an online survey was used, which corresponds to a prospective research. Similarly, it is not specified which participants were followed over time to see who did or did not develop the outcome of interest.

Answer: This study was a retrospective study using collected medical records. Since the content was web-based, it was not a prospective study using an online survey, and this is now clearly stated in the text. In addition, data on 2,504 employees who were assessed both in 2019 and 2022 was included and this was shown in Figure 3 of the results. We have revised both the methods and the results of the study.

Methods section

Before

This observational cohort study was carried out as a component of the mandatory health checkup program for employees at Juntendo University Hospital in Tokyo, Japan in 2022. The healthcare staff assessed consisted of doctors, residents, nurses, pharmacists, medical technologists, clerks, and others (e.g., nursing assistants, nutritionists, psychologists, and social workers).

After

This study is an observational cohort study that extracted the results of a web-based health checkup conducted as part of a mandatory health screening at Juntendo University Hospital in Tokyo, Japan, in 2022. The data was collected on April 10, 2023.

The target population consisted of all employees working at Juntendo University Hospital (a total of 4,786 individuals), including physicians, residents, nurses, pharmacists, clinical laboratory technicians, administrative staff, as well as other healthcare-related professionals such as nursing assistants, dietitians, psychologists, and social workers.

Regarding the sample size, of the 4,786 individuals who participated in the health checkup, valid responses were obtained from 4,700, and 4,436 individuals consented to participate in the study. No opt-out requests were recorded. Ultimately, data from the 4,436 participants were analyzed. Additionally, longitudinal analysis was conducted for 2,504 individuals who had comparable data from both 2019 and 2022.

Results section

Before

Subsequently, we analyzed the factors expected to worsen the mental state of employees during the COVID-19 pandemic. For the employees in 2022 who also had paired data of negative CES-D for those in 2019 (total 1,872), logistic regression analysis was performed using binary category as the outcome variable (i.e., CES-D positive findings in 2022), and similar factors (single regression, correlation with a P value of < .2) were selected as the dependent variables. A forest plot was created for items that were significantly associated (P < .05) (Figure 3).

After

Subsequently, we analyzed the factors expected to worsen the mental state of employees during the COVID-19 pandemic. For the employees in 2022 who also had paired data of negative CES-D for those in 2019 (total 1,872), logistic regression analysis was performed using binary category as the outcome variable (i.e., CES-D positive findings in 2022), and similar factors (single regression, correlation with a P value of < .2) were selected as the dependent variables. A forest plot was created for items that were significantly associated (P < .05) (Figure 3). Questions related to “Q5 (Stress control),” “Q4 (To feel stressed)” and “Q36 (To sleep well),” had the highest odds ratios

The validity and reliability of the measurement instruments were not specified, which may affect the quality of the research, as the results may be inconsistent or erratic.

Answer: Studies that present aggregated accuracy indices at selected cut-off points for the CES-D scale obtained using the recommended hierarchical meta-analysis methodology have shown that the CES-D has acceptable screening accuracy for detecting depression. We had added the following citations.

Vilagut G, Forero CG, Barbaglia G, Alonso J. Screening for Depression in the General Population with the Center for Epidemiologic Studies Depression (CES-D): A Systematic Review with Meta-Analysis. PLoS One. 2016;11(5):e0155431.

And we added a further note in the limitations section regarding the lack of validation for the lifestyle questionnaire.

We have revised both the introduction and the limitations of the study.

Introduction section

Before

A total of 4,239 participants completed the self-assessment using the Center for Epidemiologic Studies-Depression Scale (CES-D) [9], with a score of 16 or higher considered indicative of depression.

After

A total of 4,239 participants completed the self-assessment using the Center for Epidemiologic Studies-Depression Scale (CES-D) [9], with a score of 16 or higher considered indicative of depression. According to a study that presented pooled estimates of accuracy indices at selected cutoff points of the CES-D scale using recommended hierarchical meta-analytic methods, the CES-D demonstrates acceptable screening accuracy for detecting depression.［10］

[10] Vilagut G, Forero CG, Barbaglia G, Alonso J. Screening for Depression in the General Population with the Center for Epidemiologic Studies Depression (CES-D): A Systematic Review with Meta-Analysis. PLoS One. 2016;11(5):e0155431.

the limitations of the study

Before

The limitations of this study include its observational design and dependence on a single depression scale (CES-D). For instance, factors related to occupational stress and other psychological assessments were not fully explored. Additionally, since the lifestyle questionnaire was self-reported, the responses may have been influenced by the workers' personal biases.

After

The limitations of this study include its observational design and dependence on a single depression scale (CES-D). For instance, factors related to occupational stress and other psychological assessments were not fully explored. Additionally, since the lifestyle questionnaire was self-reported, the responses may have been influenced by the workers' personal biases. The lifestyle questionnaire used in this study has not been formally validated.

On the other hand, the results are not consistent with the purpose of the study.

The statistical processing of the data is not consistent with the objective of the study, significant differences have been made, but the objective of the study indicates that it is the association of two variables.

Answer：

Thank you very much for your valuable feedback.
The objective of this study was to examine how lifestyle changes due to the COVID-19 pandemic, particularly decreased physical activity, were associated with depressive symptoms among healthcare workers, as measured by CES-D positivity. To test this hypothesis, we compared CES-D scores, positivity rates, and lifestyle factors before (2019) and after (2022) the COVID-19 pandemic.

The statistical methods used in this study, were employed solely to visualize and quantitatively assess the association between two variables. We did not intend to investigate any causal relationships.

Statistical analyses were carried out using SPSS Statistics 29 (IBM Corp.). The chi-square test was applied to evaluate differences in the frequency of patient characteristics (e.g., gender). Clinical variables were compared using the two-tailed Mann–Whitney U test for two groups, and the Kruskal–Wallis test for three or more groups. A two-tailed P value of < .05 was deemed statistically significant for all tests.

Additionally, logistic regression analysis was used with CES-D positivity as the outcome variable and various lifestyle factors and individual characteristics (i.e., gender, age, occupation, BMI, and living alone or not) as explanatory variables, in order to evaluate the presence and strength of associations with depressive symptoms.

Therefore, we believe that the statistical approach we adopted is consistent with our original research objective of examining associations between variables.
In addition, based on the comments by the referee, we have revised the relevant sections of the methods to clarify the relationship between the research objective, analytical methods, and results.
We hope this addresses your concerns.

Methods section

Before

The CES-D was employed for self-assessment, with a score of ≥ 16 being regarded as indicative of depression. Statistical analyses were carried out using SPSS Statistics 29 (IBM Corp.). The chi-square test was applied to evaluate differences in the frequency of patient characteristics (e.g., gender). Clinical variables were compared using the two-tailed Mann–Whitney U test for two groups, and the Kruskal–Wallis test for three or more groups. A two-tailed P value of < .05 was deemed statistically significant for all tests. The relationship between participants' variables was analyzed using univariate analysis, which focused only on variables related to CES-D scores. To explore the associations between various clinical factors and depression, logistic regression analysis was performed with multiple categories as the outcome variables (i.e., CES-D positive results).Additionally, to examine the influence of each questionnaire result on the CES-D, a logistic regression analysis was conducted, considering the influence of the individual characteristics of the participants, namely 1) gender, 2) age, 3) occupation, 4) BMI, and 5) living alone/not.

After

The CES-D was employed for self-assessment, with a score of ≥ 16 being regarded as indicative of depression. Statistical analyses were carried out using SPSS Statistics 29 (IBM Corp.). The chi-square test was applied to evaluate differences in the frequency of patient characteristics (e.g., gender). Clinical variables were compared using the two-tailed Mann–Whitney U test for two groups, and the Kruskal–Wallis test for three or more groups. A two-tailed P value of < .05 was deemed statistically significant for all tests. The relationship between participants' variables was analyzed using univariate analysis, which focused only on variables related to CES-D scores. To explore the associations between various clinical factors and depression, logistic regression analysis was performed with multiple categories as the outcome variables (i.e., CES-D positive results). Additionally, to examine the influence of each questionnaire result on the CES-D, a logistic regression analysis was conducted, considering the influence of the individual characteristics of the participants, namely 1) gender, 2) age, 3) occupation, 4) BMI, and 5) living alone/not. The statistical methods used in this study, including significance testing (the chi-square test, the two-tailed Mann–Whitney U test, and the Kruskal–Wallis test) and logistic regression analysis, were employed solely to visualize and quantitatively assess the association between two variables. They were not used to investigate any causal relationships.

In the abstract the conclusion has been omitted; but it has not been omitted in the discussion.

Answer: Thank you for this important suggestion. We obtained results that support our hypothesis, such as the possibility that the impact of the "new lifestyle" on step count could affect depressive symptoms. Additionally, we were able to obtain findings that suggest a relationship between the pandemic's impact and depressive symptoms. We have revised the abstract of the study.

Abstract section

Before

When restrictions on movement are relaxed, immediate rebuilding of better lifestyle habits is necessary to maintain good mental health among healthcare workers.

After

We obtained results that support our hypothesis, such as the possibility that the impact of the "new lifestyle" on step count could affect depressive symptoms. Additionally, we were able to obtain findings that suggest a relationship between the pandemic's impact and depressive symptoms.　When restrictions on movement are relaxed, immediate rebuilding of better lifestyle habits is necessary to maintain good mental health among healthcare workers.

The manuscript is generally understandable and written in standard English, but there are syntactic and grammatical errors that should be improved to make it clearer and more precise (inconsistency in verb tenses, some sentences are too long or lack appropriate connectors, affecting fluency, some prepositions can be improved).

Answer: The conclusion was clearly stated in the abstract. In addition, this manuscript had been proofread by Enago (https://www.enago.jp/), and we had reviewed the text and made some corrections.

The ethical principles applied in the research are specified in lines 112 to 119. The manuscript does not comply with the main STROBE guidelines (in addition to what is described, it is noted that the methodological aspects are incomplete; the population, sample size, type of sampling, eligibility criteria, selection methods, follow-up methods,are not stated).

Answer: Thank you for this important suggestion. All required items have been addressed in accordance with the STROBE (Strengthening the Reporting of Observational Studies in Epidemiology) guidelines.

<https://www.equator-network.org/wp-content/uploads/2015/10/STROBE-Japanese.pdf>

<https://www.strobe-statement.org/>

Regarding the selection and follow-up methods, this study adhered to the “Ethical Guidelines for Medical Research for Humans” and the Declaration of Helsinki and the research protocol was approved by the Juntendo University School of Medicine Ethics Committee (approval no. E22-0392). As a retrospective study utilizing medical records collected after informed consent was obtained (approval no. E21-0356), an opt-out approach was implemented. Details about the study design were made available on the Juntendo University Hospital website, and all participants were given the option to opt out of the study. A total of 4,436 participants responded to the questionnaire. For the 2,504 individuals who had data available from both 2019 and 2022, a time-series analysis was also conducted.

With respect to other points, we explained in the methods section.

Methods section

Before

This observational cohort study was carried out as a component of the mandatory health checkup program for employees at Juntendo University Hospital in Tokyo, Japan in 2022. The healthcare staff assessed consisted of doctors, residents, nurses, pharmacists, medical technologists, clerks, and others (e.g., nursing assistants, nutritionists, psychologists, and social workers).

After

This study is an observational cohort study that extracted the results of a health checkup conducted as part of a mandatory health screening at Juntendo University Hospital in Tokyo, Japan, in 2022. The data was collected on April 10, 2023.

The target population consisted of all employees working at Juntendo University Hospital (a total of 4,786 individuals), including physicians, residents, nurses, pharmacists, clinical laboratory technicians, administrative staff, as well as other healthcare-related professionals such as nursing assistants, dietitians, psychologists, and social workers.

Regarding the sample size, of the 4,786 individuals who participated in the health checkup, valid responses were obtained from 4,700, and 4,436 individuals consented to participate in the study. No opt-out requests were recorded. Ultimately, data from the 4,436 participants were analyzed. Additionally, longitudinal analysis was conducted for 2,504 individuals who had comparable data from both 2019 and 2022.

As for the type of sampling, a census survey was conducted involving all employees of Juntendo University Hospital, which corresponds to a convenience sampling method. This survey was not voluntary but was conducted as part of a regular health checkup.

The eligibility criteria were staff who underwent the health checkup at Juntendo University Hospital in 2022, provided valid responses to the questionnaire, and consented to participate in the study (n=4,436). The exclusion criteria included individuals who left questions unanswered, provided incomplete responses, or opted out (none in this study).

In the discussion the results are not presented according to the study objective, the discussion is not made of the key results of the association of the two study variables (lifestyles and depressive symptoms).

Answer:

Thank you very much for your valuable feedback.

The objective of this study was to examine how the significant lifestyle changes due to the COVID-19 pandemic may have made healthcare workers, who were at the forefront of COVID-19 treatment, more susceptible to depression, and how physical inactivity, which may have resulted from behavioral restrictions, may have had a particularly strong impact on CES-D positivity. In line with this objective, our results showed that certain lifestyle factors, such as physical inactivity (e.g., Q18: "How many steps do you take on average per day?"), were associated with CES-D positivity in 2022, which suggests that the "new lifestyle" during the pandemic may have contributed to depressive symptoms among healthcare workers. The significant association between Q3 ("Do you smoke cigarettes habitually?") and CES-D positivity was also noted, and given that the increased risk of death from COVID-19 for smokers is well documented, it is plausible that smokers may have experienced heightened stress during the pandemic, potentially exacerbating depressive symptoms.

Furthermore, when examining the transition of CES-D positivity from 2019 to 2022, we found that questions related to mental health (e.g., Q5: "Stress control," Q4: "To feel stressed," Q36: "To sleep well") had the highest odds ratios for conversion to CES-D positivity. Additionally, questions related to health information (Q24–Q29) ranked among the top predictors, which may indicate the growing concern about health information and its impact on mental health. The significant role of social media during the pandemic is also noteworthy. As the prevalence of social media use increased, studies have shown that exposure to misinformation and COVID-19-related news on social media could contribute to heightened anxiety and depression. We hypothesize that the use of social media during the pandemic may have worsened emotional and mental health, potentially increasing the susceptibility to depressive symptoms among healthcare workers.

In summary, the findings of this study suggest that lifestyle changes during the COVID-19 pandemic, particularly physical inactivity, smoking, and increased exposure to information through social media, contributed to depressive symptoms among healthcare workers. These results align with the study's objective of examining how these factors influenced CES-D positivity and support the initial hypothesis. We have revised the discussion of the study.

Discussion section

Before

Thus, staff members who are confused about any health information on social media should be educated on this matter. When restrictions on movement have been relaxed, immediate rebuilding of better lifestyle habits is necessary to maintain good mental health.

After

Thus, staff members who are confused about any health information on social media should be educated on this matter.

In summary, the findings of this study suggest that lifestyle changes during the COVID-19 pandemic, particularly physical inactivity, smoking, and increased exposure to information through social media, contributed to depressive symptoms among healthcare workers. These results align with the study's objective of examining how these factors influenced CES-D positivity and support the initial hypothesis. When restrictions on movement have been relaxed, immediate rebuilding of better lifestyle habits is necessary to maintain good mental health.

In addition, no data available at the beginning of the PDF file of the manuscript are included, only inclusion of figures is mentioned.

Answer: Research data are not shared. The raw data belonged to the present study cannot be made publicly available because the disclosure of personal data was not included in the research protocol of the present study. The data are not publicly available due to privacy and ethical restrictions. This is explained in the Data availability statement.

Data availability statement:

Research data are not shared. The raw data belonged to the present study cannot be made publicly available because the disclosure of personal data was not included in the research protocol of the present study. The data are not publicly available due to privacy and ethical restrictions.
